# P-502. Incorporating Behavioral Economics to Educate Adolescents and Young Adults about HIV Pre-Exposure Prophylaxis

**DOI:** 10.1093/ofid/ofae631.701

**Published:** 2025-01-29

**Authors:** Carly Guss, Brittany Gluskin, Douglas Krakower, Lauren Wisk

**Affiliations:** Boston Children's Hospital, Boston, Massachusetts; Boston Children's Hospital, Boston, Massachusetts; Harvard Medical School, Boston, MA; University of California, Los Angeles, Los Angeles, California

## Abstract

**Background:**

Despite disproportionately high rates of HIV diagnoses, knowledge about and interest in pre-exposure prophylaxis (PrEP) is low among adolescents and young adults (AYA) in the USA. AYA need PrEP educational strategies that are tailored to their developmental stage. We hypothesized that behavioral economics techniques could help optimize PrEP messaging.

**Methods:**

The research team developed two brief social media-style educational videos on PrEP. Both videos reviewed the same facts, but one used gain-framed messaging (emphasized positive effects of using PrEP) while the other featured loss-framed messaging (emphasized negative consequences of non-use). Thirty AYA were shown the videos and interviewed about their overall impressions, opinions on facts presented, message framing preferences, and impact (e.g., what steps they might take after viewing). The deidentified interview transcripts were reviewed for emerging themes by two coders until consensus was reached.

**Results:**

Interviews were held May – October 2023. Participants ranged in age from 15-25 years (mean 21.5 years, IQR 17-23.8 years) and identified as White (70.0%) or Black/African American (13.2%). Most (80.0%) identified as cisgender and 16.7% as transgender. A third (33.3%) had ever tested for HIV, none had an HIV diagnosis, and 36.7% did not feel that PrEP applied to them (i.e., viewed themselves as low risk for HIV). Thematic findings included: participants generally expressed positive views of PrEP, would like to see the video in a doctor’s office, and viewing the video would lead them to do their own research. Gain-framed messaging was preferred over the loss-framed video messaging, though some youth felt the videos were too similar. PrEP efficacy was the most important medical fact presented. Youths disliked advertisement-like or “staged” video elements and favored genuineness and relatability.

**Conclusion:**

AYA preferred gain-framed over loss-frame PrEP messaging, and information on PrEP efficacy delivered by authentic messengers is likely to resonate. These findings offer novel insights into AYA perspectives around PrEP messaging for brief videos that could be disseminated through social media for PrEP education, which could help empower AYA to make informed decisions around PrEP as part of their sexual health.

**Disclosures:**

**Douglas Krakower, MD**, FluidForm Bio: Stocks/Bonds (Private Company)|Gilead: Grant/Research Support|Matchbox Health: Stocks/Bonds (Private Company)|Medscape: Funding to develop educational content|Merck: Grant/Research Support

